# *Helicobacter pylori* induces somatic mutations in *TP53* via overexpression of CHAC1 in infected gastric epithelial cells

**DOI:** 10.1002/2211-5463.12402

**Published:** 2018-03-09

**Authors:** Yuriko Wada, Kosuke Takemura, Padmaja Tummala, Keisuke Uchida, Keisuke Kitagaki, Asuka Furukawa, Yuuki Ishige, Takashi Ito, Yukichi Hara, Takashige Suzuki, Hitomi Mimuro, Philip G. Board, Yoshinobu Eishi

**Affiliations:** ^1^ Department of Human Pathology Graduate School and Faculty of Medicine Tokyo Medical and Dental University Japan; ^2^ Department of Urology Tokyo Metropolitan Cancer and Infectious Diseases Center Komagome Hospital Japan; ^3^ The ACRF Department of Cancer Biology and Therapeutics Group of Molecular Genetics The John Curtin School of Medical Research Australian National University Canberra ACT Australia; ^4^ Division of Surgical Pathology Tokyo Medical and Dental University Hospital Japan; ^5^ Department of Infection Microbiology Division of Infectious Diseases Research Institute for Microbial Diseases University of Osaka Japan

**Keywords:** *cagA*, CHAC1, glutathione, *H. pylori*, p53, ROS

## Abstract

Infection with *Helicobacter pylori* is known to decrease the level of glutathione in gastric epithelial cells and increase the production of reactive oxygen species (ROS), which can lead to DNA damage and the development of gastric cancer. Cation transport regulator 1 (CHAC1) has γ‐glutamylcyclotransferase activity that degrades glutathione. We found that *cagA*‐positive *H. pylori* infection triggered CHAC1 overexpression in human gastric epithelial (AGS) cells leading to glutathione degradation and the accumulation of ROS. Nucleotide alterations in the *TP53* tumour suppressor gene were induced in AGS cells overexpressing CHAC1, whereas no mutations were detected in cells overexpressing a catalytically inactive mutant of CHAC1. A high frequency of *TP53* mutations occurred in *H. pylori*‐infected AGS cells, but this was prevented in cells transfected with CHAC1 siRNA. These findings indicate that *H. pylori*‐mediated CHAC1 overexpression degrades intracellular glutathione, allowing the accumulation of ROS which subsequently causes mutations that could contribute to the development of gastric cancer.

**Abbreviations**
ACTB: β‐actin
AGS cells: human gastric epithelial cells
CHAC1: cation transport regulator 1
ER: endoplasmic reticulum
GSH: glutathione
*H. pylori*: *Helicobacter pylori*
ROS: reactive oxygen species

It is well established that *Helicobacter pylori (H. pylori)* infection can increase the production of reactive oxygen species (ROS) and decrease the level of glutathione (GSH) in gastric epithelial cells 1, 2, 3. These changes in redox balance and the elevation of oxidative stress lead to mutations in DNA that potentially contribute to the development of gastric carcinoma 4, 5. GSH provides the reducing equivalents used in the protection of cellular macromolecules against the oxidative damage caused by ROS 6, 7. However, it is not clear from prior studies whether the decrease in GSH associated with *H. pylori* infection results directly from its consumption following the excessive production of ROS, or the accumulation of ROS is exacerbated by a deficiency of GSH.

Recent studies have shown that a protein known as cation transport regulator 1 (CHAC1) is a novel member of the γ‐glutamylcyclotransferase family of enzymes that contribute to the γ‐glutamylcycle 8, 9. CHAC1 can catalyse the cleavage of GSH into 5‐oxoproline and the dipeptide cysteinylglycine and is one of the only cytosolic enzymes known to degrade GSH 8, 9. Overexpression of CHAC1 leads to GSH depletion and a change in the cellular redox balance 8, 9. CHAC1 has also been identified as a component of the unfolded protein response stress signalling pathway in the endoplasmic reticulum (ER) 10, 11, and its elevated mRNA expression level has been associated with a poor outcome in patients with breast and ovarian cancer 12, 13.

Given the role of CHAC1 in the degradation of GSH, we tested the hypothesis that the changes in GSH levels observed in *H. pylori*‐infected cells result from the induction of CHAC1. We report here that *H. pylori* infection causes increased expression of CHAC1 that leads to GSH depletion, elevated ROS accumulation and increased somatic DNA mutations in the *TP53* tumour suppressor gene.

## Materials and methods

### Bacteria and infection

A standard strain (43 504) of *H. pylori* (*cagA*‐positive *H. pylori*) was purchased from American Type Culture Collection (ATCC; Manassas, VA, USA). An isogenic *cagA‐*knockout *H. pylori* mutant (43 504) (*cagA*‐negative *H. pylori*) was kindly provided by one of the authors (HM). Prior to infection, *H. pylori* was grown in Brucella broth (Becton Dickinson, Sparks, MD, USA) with 10% fetal bovine serum (FBS) for 24 h.

For infection, *H. pylori* was added to human gastric epithelial (AGS) cells at a ratio of 100 bacteria per cell.

For the *TP53* mutation analysis, AGS cells were infected with either *cagA‐*positive or *cagA*‐negative *H. pylori* every 3 days for 15 days postinfection. Before each reinfection, the cells were reseeded at a rate of 2 × 10^5^ cells/well. Samples for CHAC1, GSH, ROS and mutation analysis were taken on day 16 after the first infection.

### Cell culture and transfection

AGS cells obtained from ATCC were grown in RPMI‐1640 (Sigma‐Aldrich Co., St. Louis, MO, USA) supplemented with 10% FBS, 100 U·mL^−1^ penicillin and 100 μg·mL^−1^ streptomycin. Lipofectamine 2000 reagent (Invitrogen, Carlsbad, CA, USA) was used for transfection of the plasmids and small interfering RNA (siRNA). A wild‐type CHAC1 cDNA clone in the expression vector pCMV6 (CHAC1‐WT) was obtained from OriGene Technologies (Rockville, MD, USA), and an inactive mutant (CHAC1‐MT) was created by the insertion of an E157Q substitution in the active site.

For CHAC1 knockdown, cells were treated with synthesized siRNA (5′‐AUCUUCAAGGAGCGUCACCAC‐3′). An unrelated scrambled siRNA (5′‐GUUAAAUAGCGAUAGGAAUUC‐3′) was used as a control for non‐sequence‐specific effects. AGS cells were transfected with a final working concentration of 50 nmol·L^−1^ siRNA in original RPMI‐1640 at 6 h before *H. pylori* infection.

### RNA extraction and real‐time RT‐PCR

AGS cells were treated for RNA extraction with 1.0 mL of TRIzol reagent (Invitrogen) according to the manufacturer's instructions, and cDNA was synthesized with random primers using Superscript III Reverse Transcriptase (Invitrogen). Oligonucleotide primers and probes are listed in Table S1. Relative quantitation of mRNA was performed by real‐time reverse transcription (RT)‐PCR using TaqMan Universal PCR Master Mix (ABgene, Epsom, UK). Amplification and detection were performed with the ABI PRISM 7900HT Sequence Detection System (Applied Biosystems, Foster City, CA, USA).

### Measurement of the intracellular GSH concentration

The intracellular GSH concentration was measured as indicated by the manufacturer's instructions (GSSG/GSH Quantification Kit; Sigma‐Aldrich Co.). A standard line was drawn using GSSG solution at various concentrations.

### ROS measurement

The cells were washed twice with PBS, and the media were changed to original RPMI‐1640 containing a final working concentration of 20 μmol·L^−1^ carboxy‐H_2_DCFDA (Invitrogen) dissolved in dimethylsulfoxide (Wako Pure Chemical Industries, Ltd., Osaka, Japan). For a negative control, 20 μL of dimethylsulfoxide was added to the media. After incubation, the cells were trypsinized, washed and resuspended with PBS for examination by flow cytometry (FACSCanto II; Becton Dickinson).

### Production of anti‐CHAC1 antibody

A full‐length human CHAC1 cDNA was subcloned in the pHUE vector, and recombinant protein was expressed in *Escherichia coli* and purified as described previously 14. A novel anti‐CHAC1 monoclonal antibody (IgM, κ) termed CHAC1‐mAb_(v1v2)_ was made for the study by immunizing BALB/c mice (CLEA Japan, Tokyo, Japan) with the purified recombinant human CHAC1 protein, and hybridoma cell lines were prepared according to the previously described protocol 15. Hybridoma cell lines producing anti‐CHAC1 antibodies were verified by enzyme‐linked immunosorbent assay with the recombinant CHAC1 protein and were characterized further by immunohistochemistry and immunoblotting of CHAC1 expressed in transfected HEK293T cells purchased from ATCC (Fig. S1).

### Western blotting

For protein extraction, AGS cell lysates were harvested with M‐PER Mammalian Protein Extraction Reagent (Thermo Fisher Scientific Inc., Kanagawa, Japan) supplemented with protease inhibitor cocktail (Sigma‐Aldrich Co.). Western blotting was performed as previously described 16 with minor modifications. The membranes were probed with the appropriately diluted primary antibodies (CHAC1‐mAb_(v1v2)_ or anti‐β‐actin antibody [#4970; Cell Signaling Technology, Beverly, MA, USA]), followed by incubation with the appropriate secondary antibodies. Blots were developed with ECL (Bio‐Rad, Hercules, CA, USA). Finally, images were recorded and analysed with ChemiDoc MP Image System (Bio‐Rad).

### Sequence analysis of *TP53*

AGS cells were treated with 1.0 mL of TRIzol reagent for RNA extraction, and cDNA was synthesized as described above. A total of 1179 bp of exome sequence between exons 2 and 11 of *TP53* was amplified with PrimeSTAR Max DNA Polymerase (Takara Shuzo, Shiga, Japan) using the primers listed in Table S1. PCR products were subcloned using the TOPO TA Cloning Kit (Invitrogen) according to the manufacturer's instructions, followed by analysis of nucleotide sequences in randomly selected clones with ABI BigDye terminator ver. 3.1 (Applied Biosystems) and ABI Prism 3130xl Genetic Analyzer (Applied Biosystems).

### Statistical analysis

GraphPad PRISM ver. 6 (GraphPad Software, Inc., CA, USA) was used for statistical analysis. The results are expressed as means ± standard errors of the means (SEM). A two‐sided *P *<* *0.05 was regarded as statistically significant. A two‐way analysis of variance (ANOVA) and further analysis using Tukey's multiple comparisons test were used to test for statistical significance of the time‐independent mRNA expression of CHAC1. A one‐way analysis of variance (ANOVA) and further analysis using Holm–Sidak's multiple comparisons test and unpaired *t*‐test were used to test for comparison of intracellular levels of CHAC1, GSH and ROS. The *TP53* mutation frequencies were analysed by Fisher's exact test.

## Results

### *H. pylori* induces CHAC1 expression, GSH depletion and ROS accumulation in infected cells

**Figure 1:**
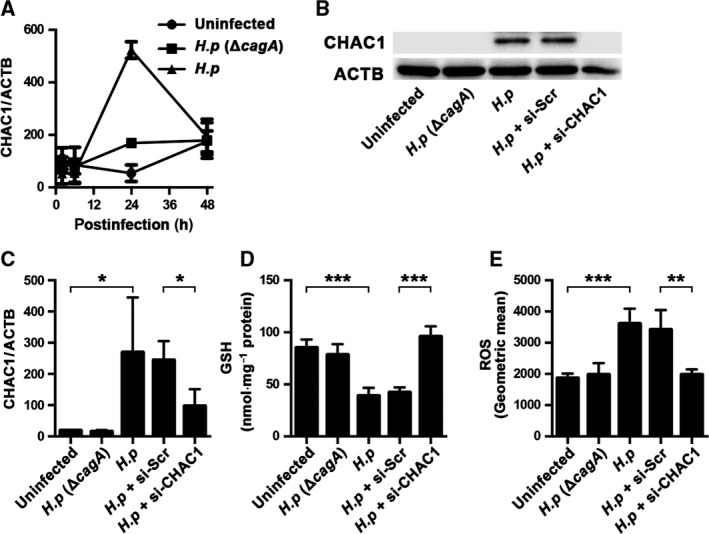
*H. pylori* infection and CHAC1 expression. (A) CHAC1 mRNA expression normalized with that of β‐actin (ACTB) in AGS cells was measured after *cagA*‐positive or *cagA*‐negative *H. pylori* infection. Total RNA was isolated at 2, 6, 24 and 48 h postinfection. (B–E) Untreated AGS cells were infected by either *cagA*‐positive or *cagA*‐negative *H. pylori* for 24 h. AGS cells transfected with CHAC1 siRNA or scrambled siRNA were also infected by *cagA*‐positive *H. pylori*. (B) The protein expression of CHAC1 and ACTB; (C) the mRNA expression of CHAC1 normalized with ACTB; (D) the level of intracellular GSH; (E) the level of intracellular ROS. *H.p* (Δ*cagA*) indicates AGS cells infected with *cagA*‐negative *H. pylori; H.p* indicates cells infected with *cagA*‐positive *H. pylori; H.p *+ si‐Scr indicates cells infected with *cagA*‐positive *H. pylori* with transfection of scrambled siRNA;* H.p *+ si‐CHAC1 indicates cells infected with *cagA*‐positive *H. pylori* with transfection of siRNA CHAC1. Data shown for A and C are the mean ± SE from triplicate measurements derived from three replicate experiments, and data for D and E are the mean ± SE from triplicate measurements derived from four replicate experiments. *P <* *0.05, **P <* *0.01 and ***P <* *0.001.

AGS cells infected by *cagA*‐positive *H. pylori* significantly (*P *<* *0.0001) expressed CHAC1 mRNA with peak expression at 24 h postinfection (Fig. 1A). In contrast, CHAC1 was not detected in uninfected AGS cells and was not strongly induced by *cagA*‐negative *H. pylori*. At 24 h postinfection, intracellular levels of CHAC1 (mRNA and protein), GSH and ROS were measured in *cagA*‐positive *H. pylori‐*infected AGS cells transfected with CHAC1 siRNA or scrambled control siRNA (Fig. 1B–E). Infection of AGS cells with *cagA*‐positive *H. pylori* induced a significant (*P *<* *0.05) > 10‐fold increase in CHAC1 mRNA and protein expression (Fig. 1B,C). At the same time, there was a significant (*P *<* *0.001) decrease in GSH (Fig. 1D) and a significant (*P *<* *0.001) increase in ROS (Fig. 1E). Moreover, no changes in the GSH (Fig. 1D) and ROS (Fig. 1E) levels were observed in the *cagA*‐positive *H. pylori*‐infected AGS cells when the CHAC1 expression was suppressed by siRNA transfection to a level that was undetectable by western blotting (Fig. 1B). These experiments show that *cagA*‐positive *H. pylori* infection induces CHAC1 which degrades GSH thereby allowing ROS to accumulate.

### Overexpression of catalytically active CHAC1 is required for the induction of *TP53* mutations in the *H. pylori*‐infected cells

**Figure 2:**
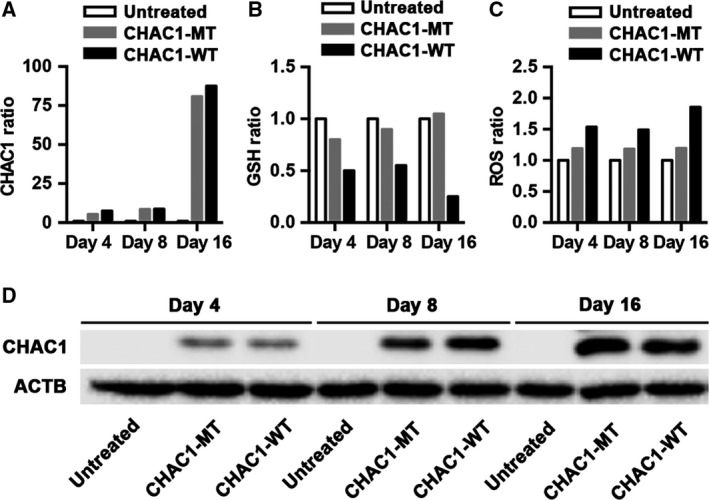
Levels of CHAC1 mRNA, GSH, ROS and CHAC1 protein in CHAC1‐transfected samples used for mutation analysis. (A–D) AGS cells expressing CHAC1‐WT or inactive CHAC1‐MT and untreated cells were cultured for 4, 8 and 16 days. The values of CHAC1 mRNA, GSH and ROS are the mean of two replicates used for mutation analysis and are expressed as the ratio of treated to untreated values.

To confirm the observation that the effects of CHAC1 expression on GSH depletion and ROS accumulation are due to the enzymatic activity of CHAC1, we transfected AGS cells with wild‐type (CHAC1‐WT) and a catalytically inactive mutant CHAC1 (CHAC1‐MT). After 16 days, CHAC1‐WT and CHAC1‐MT (mRNA and protein) were expressed at similar levels (Fig. 2A,D). At this time, there was a decrease in GSH and an increase in ROS levels in cells expressing catalytically active CHAC1‐WT (Fig. 2B,C). In contrast, there was no change in the levels of GSH or ROS in transfected cells expressing inactive CHAC1‐MT (Fig. 2B,C).

To determine whether CHAC1 expression resulted in an increased mutation rate, a mutation analysis was undertaken on the untransfected and transfected cells after 4, 8 and 16 days. Nucleotide alterations in DNA recovered from these AGS cells were determined in the sequence between exons 2 and 11 of the tumour suppressor gene *TP53*. No mutations were detected in *TP53* in untransfected control AGS cells or in cells transfected with catalytically inactive CHAC1‐MT (Table 1). In contrast, cells transfected with catalytically active CHAC1‐WT had multiple mutations that encoded amino acid substitutions in the *TP53* gene. The frequency of these mutations increased in a time‐dependent manner (Table 1).

**Table 1:** *TP53* mutation in AGS cells related to CHAC1 overexpression

	Total mutated clones	Mutations causing amino acid substitutions	*P* value*
AGS cells with CHAC1 overexpression by transfection			
Day 4 CHAC1‐WT	5/60	2/70 740 (0.28/10^4^)	N.S.
Day 8 CHAC1‐WT	10/59	7/69 561 (1.01/10^4^)	0.0074
Day 16 CHAC1‐WT	20/59	13/69 561 (1.87/10^4^)	0.0001
Day 16 CHAC1‐MT	0/50	0/58 950 (0.00/10^4^)	N.S.
Day 16 control	0/60	0/70 740 (0.00/10^4^)	–
AGS cells with CHAC1 overexpression induced by *H. pylori* infection			
Uninfected	0/60	0/70 740 (0.00/10^4^)	–
*cagA* (−) *H. pylori*	0/59	0/69 561 (0.00/10^4^)	N.S.
*cagA* (+) *H. pylori*	14/59	8/69 561 (1.15/10^4^)	0.0037
*cagA* (+) *H. pylori* + scrambled siRNA	10/53	6/62 487 (0.96/10^4^)	0.0107
*cagA* (+) *H. pylori* + CHAC1 siRNA	0/58	0/68 382 (0.00/10^4^)	N.S.

*P values for the number of mutated bases causing amino acid substitutions were evaluated by Fisher's exact test.

Total mutated clones indicates the total number of mutations per total number of clones examined; Mutations causing amino acid substitutions indicates the number of mutated bases causing amino acid substitutions per total number of base pairs sequenced. Frequency of mutations causing amino acid substitutions per 10^4^ base pairs is shown in parentheses. AGS cells were subjected to *H. pylori* infection for 16 days (see Methods). N.S., not significant.

To extend this study, we next performed the same *TP53* mutation analysis on *H. pylori*‐infected AGS cells transfected with either CHAC1 siRNA or scrambled control siRNA. The levels of CHAC1 mRNA, GSH, ROS and CHAC1 protein in samples used for the mutation analysis are shown in Fig. S2A–D and had a similar phenotype to the infected cells shown in Fig. 1. As observed in the CHAC1‐transfected cells, *cagA*‐positive *H. pylori*‐infected cells and the infected cells transfected with scrambled control siRNA had a significantly higher frequency of nucleotide alterations in *TP5*3 than uninfected cells. In contrast, there were no mutations in *cagA*‐positive *H. pylori*‐infected cells in which CHAC1 expression was knocked down by CHAC1 siRNA (Table 1). The results of the mutation analysis presented here are the combination of two independent experiments (Tables S2 and S3). Nucleotide alterations in *TP53* causing amino acid substitutions are summarized in Table S4.

## Discussion

CHAC1 is a novel ER stress‐inducible gene first identified in human aortic endothelial cells treated with oxidized phospholipids 10, and various stimuli that trigger ER stress, including infection, upregulate CHAC1 mRNA expression 11, 17. *H. pylori*‐triggered ER stress has been reported since 2013 18, 19, 20, and the present study is the first to demonstrate that CHAC1 overexpression is induced by the *H. pylori*‐triggered ER stress.

*H. pylori* infection is known to cause the depletion of cellular GSH levels with a concomitant accumulation of ROS 1, 2, 3, 21, 22, 23, 24, 25, 26. The mechanism of *H. pylori*‐induced GSH depletion, however, remains unknown. Because CHAC1 was reported in 2012 to be a novel member of the γ‐glutamylcyclotransferase family that degrades GSH 8, we proposed that the decreased level of GSH in *H. pylori*‐infected cells may be caused by CHAC1 expression. The experiments in the present study confirmed that *cagA‐*positive but not *cagA*‐negative *H. pylori* infection induced CHAC1 overexpression. The depletion of GSH in *cagA*‐positive *H. pylori*‐infected cells was found to be due to the expression of CHAC1 as the GSH depletion did not occur in infected cells when the expression of CHAC1 was knocked down by specific CHAC1 siRNA. In addition, the expression of CHAC1 with an inactivating mutation (CHAC1‐MT) also prevented the depletion of GSH. The present results also support the view that the accumulation of ROS occurs as a result of the depletion of GSH as ROS did not accumulate in *H. pylori*‐infected cells that were transfected with CHAC1 siRNA that blocked the expression of CHAC1 protein. This result confirms the importance of GSH in the maintenance of the cells redox balance and its role in the protection of the cell against the deleterious effects of oxidative stress.

ROS interact directly with macromolecules, including genomic DNA, thereby causing damage to specific genes responsible for cell proliferation 27, 28 and tumour suppression that can lead to tumorigenesis 29. An accumulation of ROS in gastric epithelial cells infected by *H. pylori* may lead to somatic cell DNA mutations that interfere with both the expression and function of tumour‐suppressing genes such as *TP53*, and contribute to the development of gastric cancer 4, 22, 30, 31, 32. Thus, our finding that *H. pylori*‐induced CHAC1 expression is responsible for the depletion of GSH and the accumulation of intracellular ROS led us to investigate whether elevated CHAC1 expression is associated with an increased frequency of DNA mutations in infected gastric epithelial cells. Although we would expect that all DNA would be subject to oxidative damage, we focussed on the *TP53* gene because of its well‐established role as a tumour suppressor.

As expected, *TP53* mutations causing amino acid substitutions were found to increase in a time‐dependent manner in AGS cells overexpressing wild‐type CHAC1. However, no mutations were detected in untreated control cells or in cells overexpressing catalytically inactive CHAC1‐MT. The levels of CHAC1, GSH and ROS in the cells used for the mutation analysis clearly demonstrated that independent of *H. pylori* infection, CHAC1‐induced depletion of GSH and the accumulation of intracellular ROS are required for the induction of *TP53* mutations. In subsequent experiments with *H. pylori* infection, *TP53* mutations were observed in the untreated or scrambled siRNA‐transfected control cells infected by *cagA*‐positive *H. pylori*, whereas *TP53* mutations were not found in similarly infected cells transfected with CHAC1 siRNA. These experiments indicated that CHAC1 overexpression is a significant cause of *TP53* mutations in gastric epithelial cells infected by *cagA*‐positive *H. pylori*. Our view that the increase in ROS in *cagA*‐positive *H. pylori*‐infected cells leads to oxidative DNA modification is further supported by the prior observations that the occurrence of mutations and carcinogenic transformation in *H. pylori* infection are negatively correlated with the concentration of the ROS scavenger vitamin C 33.

The frequency of *TP53* mutations with amino acid substitutions in *H. pylori*‐infected AGS cells was reported in a previous study in connection with aberrant expression of activation‐induced cytidine deaminase (AID) 32. The mean mutation frequency of AGS cells caused by *H. pylori* was higher in the previous study than in the present study (2.23 vs 1.15 per 10^4^ nucleotides). This may be due to differences in the strains of *H. pylori* used for infection (a clinical isolate vs ATCC 43504) and the DNA polymerase used for sequence analysis of *TP53* (High Fidelity DNA Polymerase from Finnzymes vs PrimeSTAR Max DNA Polymerase). In the previous study, *TP53* mutations were observed even in the AID knockdown cells infected by *cag* pathogenicity island (PAI)‐positive *H. pylori* at a still high frequency (2.08 per 10^4^ nucleotides), suggesting that *H. pylori*‐mediated molecular events other than aberrant AID expression contributed to induce *TP53* mutations. Thus, the *TP*53 mutation frequencies in the two different studies performed with AID and CHAC1, respectively, suggest that potential AID‐mediated *TP53* mutations added to the basal level of CHAC1‐mediated *TP53* mutations, and both are induced by *cagA*‐positive *H. pylori* infection and may contribute to gastric carcinogenesis independently via different pathways.

**Figure 3:**
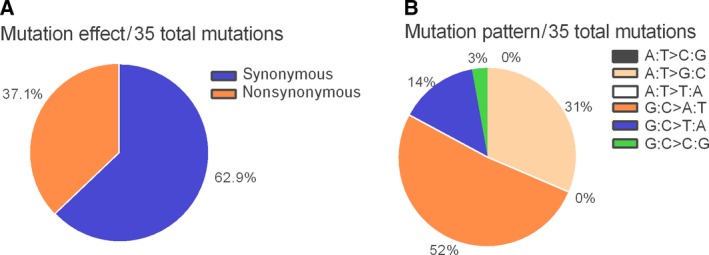
Type of somatic *TP53* mutations found in AGS cells with CHAC1 overexpression by transfection. (A) Pie charts showing the proportion of nonsynonymous and synonymous mutations. (B) The proportion of different mutation patterns of 35 total mutations.

**Figure 4:**
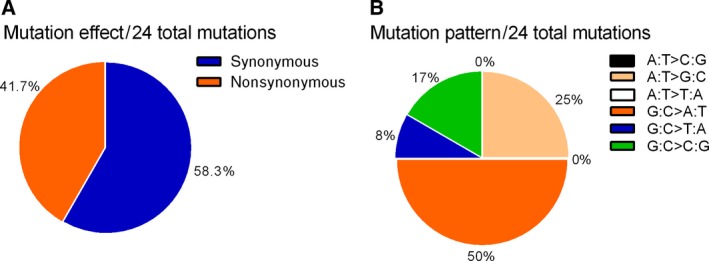
Type of somatic *TP53* mutations found in AGS cells with CHAC1 overexpression induced by *H. pylori* infection. (A) Pie charts showing the proportion of nonsynonymous and synonymous mutations. (B) The proportion of different mutation patterns of 24 total mutations.

Evaluation of *TP53* mutations in human cancer cases indicates that the majority are G : C > T:A transversions and G : C > A : T transitions 34 that are considered to result from oxidative stress and the ROS‐mediated DNA damage 35, 36. In the present study, we also noted the majority of mutations (63%, 58%) are G : C > T : A transversions and G : C > A : T transitions (Figs 3 and 4). This further supports our contention that CHAC1 expression leads ultimately to oxidative damage to DNA.

In a review of all cancer‐associated *TP53* mutations, 86% were found between codons 125 and 300, a region that corresponds to the DNA binding domain. In the present study, 47% of the mutations causing amino acid substitutions occurred within this region and are therefore more likely to be deleterious 34. Substitutions at codons 175, 245, 248, 249, 273 and 282 account for approximately 30% of cancer‐associated *TP53* mutations 34. In the present study, we detected mutations at codons 245 and 248. Together, these substitutions account for approximately 11% of previously described *TP53* cancer‐associated mutations. In addition, several mutations identified in this study at codons 176, 177, 244 and 270 are in close proximity to high‐frequency cancer‐associated codons.

In conclusion, this study has shown that *cagA*‐positive *H. pylori*‐mediated CHAC1 overexpression causes GSH depletion and the accumulation of ROS that subsequently leads to nucleotide alterations in the DNA of the infected cells. Oxidative DNA damage caused by *H. pylori*‐induced CHAC1 overexpression in infected gastric epithelial cells may directly contribute to the development of gastric cancer.

## Author contributions

YW designed the study design, performed most of the experiments, analysed and interpreted the data and wrote the manuscript. KT performed the analysis of CHAC1 expression induced by *H. pylori* infection and contributed to the study design. PT, KU, KK, AF, YI and TI provided the study material and technical support. YH conducted the mutation analysis. TS conducted the experiments. HM kindly provided the isogenic *cagA‐*knockout *H. pylori* mutant. PGB and YE supervised and directed the project and contributed to the manuscript preparation.

## Supporting information


**Fig. S1.** Reactivity of the novel monoclonal antibody to CHAC1.
**Fig. S2.** The levels of CHAC1 mRNA, GSH, ROS and CHAC1 protein in *H. pylori*‐infected samples used for mutation analysis.
**Table S1.** Primers and probes used for this study.
**Table S2.** CHAC1 overexpression leading to *TP53* mutation in AGS cells (results from each of the two experiments).
**Table S3.** CHAC1 expression induced by *H. pylori i*nfection leading to *TP53* mutation in AGS cells (results from each of the two experiments).
**Table S4.** Distribution of nucleotide alterations in the *TP53* sequence and resultant amino acid substitutions.
