# Supplementary material for: Helicobacter pylori induces somatic mutations in TP53 via overexpression of CHAC1 in infected gastric epithelial cells
Source: FEBS Open Bio. 2018 Mar 9;8(4):671–9. doi: 10.1002/2211-5463.12402 (PMC5881537; doi:10.1002/2211-5463.12402)
Supplement: Supplementary file 1 — Fig. S1. Reactivity of the novel monoclonal antibody to CHAC1. Fig. S2. The levels of CHAC1 mRNA, GSH, ROS and CHAC1 protein in H. pylori‐infected samples used for mutation analysis. Table S1. Primers and probes used for this study. Table S2. CHAC1 overexpression leading to TP53 mutation in AGS cells (results from each of the two experiments). Table S3. CHAC1 expression induced by H. pylori infection leading to TP53 mutation in AGS cells (results from each of the two experiments). Table S4. Distribution of nucleotide alterations in the TP53 sequence and resultant amino acid substitutions. [file FEB4-8-671-s001.pdf]

Supporting information

Helicobacter pylori induces somatic mutations in *TP53* via overexpression of CHAC1 in infected gastric epithelial cells

Yuriko Wada, Kosuke Takemura, Padmaja Tummala,

Keisuke Uchida, Keisuke Kitagaki, Asuka Furukawa, Yuuki Ishige, Takashi Ito, Yukichi Hara, Takashige Suzuki, Hitomi Mimuro, Philip G. Board & Yoshinobu Eishi

Fig. S1. Reactivity of the novel monoclonal antibody to CHAC1.

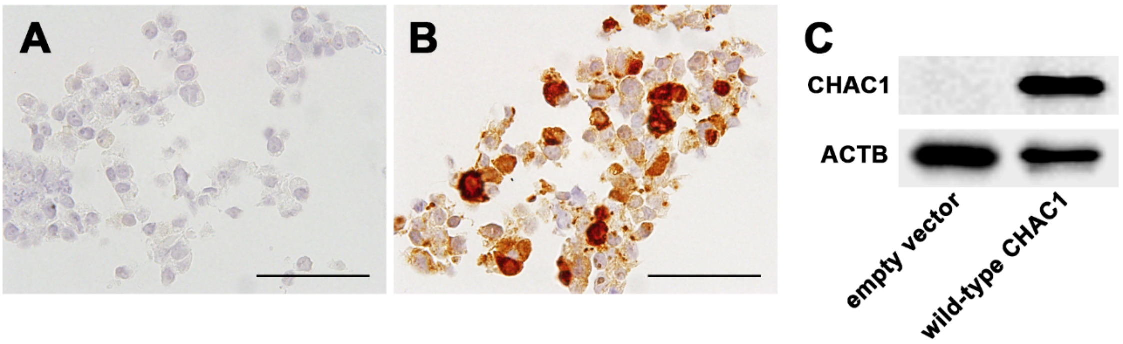

Fig. S1. (A,B) Immunohistochemistry with the CHAC1-mAb$_{(v1v2)}$ on HEK293T cells transfected with either an empty plasmid vector as a control (A) or the wild-type CHAC1 vector (B). (C) Western blotting with CHAC1-mAb$_{(v1v2)}$ or anti-β-actin (ACTB) antibody for proteins extracted from each transfected HEK293T cell culture. Bars: 50 μm.

Fig. S2. The levels of CHAC1 mRNA, GSH, ROS and CHAC1 protein in *H. pylori* infected samples used for mutation analysis.

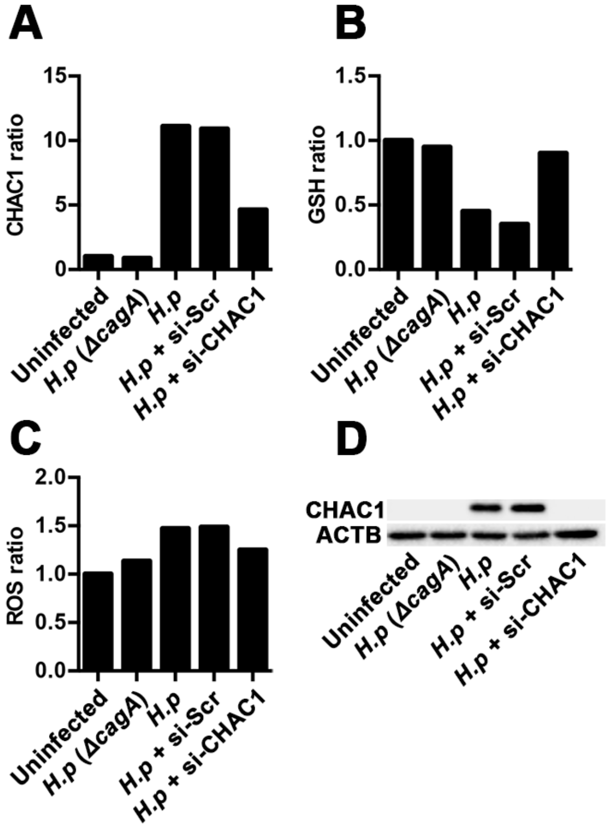

(A-D) AGS cells were infected with either *cagA*-positive or *cagA*-negative *H. pylori* every 3 days for 15 days and samples were collected for analysis on day 16 (see methods). AGS cells transfected with CHAC1 siRNA or scrambled siRNA were also infected by *cagA*-positive *H. pylori*. The values of CHAC1 mRNA, GSH and ROS are the mean of two replicates used for mutation analysis and are expressed as the ratio of uninfected to infected values. *H.p* (△ *cagA*) indicates AGS cells infected with *cagA*-negative *H. pylori; H.p* indicates cells infected with *cagA*-positive *H. pylori; H.p* + si-Scr indicates cells infected with *cagA*-positive *H. pylori* with transfection of scrambled siRNA; *H.p* + si-CHAC1 indicates cells infected with *cagA*-positive *H. pylori* with transfection of siRNA CHAC1.

Table S1. Primers and probes used for this study

Primers	Orientation	Sequence (5' – 3')
human *CHAC1*	Sense	GGTGACGCTCCTTGAAGATCAT
	Antisense	TCAGTGGTTGGTCAGGAGCAT
	Probe	[FAM]-AGAGGCAGTGCTTGGTGGCTACGATACC-[TAMRA]
human *β-actin*	Sense	GGATGCAGAAGGAGATCACTG
	Antisense	CGATCCACACGGAGTACTTG
	Probe	[TET]-CCCTGGCACCCAGCACAATG-[TAMRA]
human *TP53*	Sense	CTGCTGGGCTCCGGGGACAC
	Antisense	GTTCAGTGGAGCCCCGGGACA
human *TP53*	Sense	ACACTTTGCGTTCGGGCTG
(sequencing analysis)	Antisense	TGGAAGTCCTGGGTGCTTC

Table S2. CHAC1 overexpression leading to *TP53* mutation in AGS cells (Results from each of the two experiments)

	Results from each of the two experiments			
	1st experiment		2nd experiment	
	Total Mutated clones	Mutations causing amino acid substitutions	Total Mutated clones	Mutations causing amino acid substitutions
Day4 CHAC1-WT	2 / 30	1 / 35370 (0.28 / 10^4)	3 / 30	1 / 35370 (0.28 / 10^4)
Day8 CHAC1-WT	2 / 29	1 / 34191 (0.29 / 10^4)	8 / 30	6 / 35370 (1.70 / 10^4)
Day16 CHAC1-WT	10 / 25	6 / 29475 (2.04 / 10^4)	10 / 34	7 / 40086 (1.75 / 10^4)
Day16 CHAC1-MT	0 / 25	0 / 29475 (0.00 / 10^4)	0 / 25	0 / 29475 (0.00 / 10^4)
Day16 control	0 / 30	0 / 35370 (0.00 / 10^4)	0 / 30	0 / 35370 (0.00 / 10^4)

Exome sequence between exon 2 and 11 of *TP53* was amplified and cloned from CHAC1-WT transfected AGS cells after 4, 8 and 16 days or from CHAC1-MT transfected cells and untreated control cells after 16 days. Nucleotide sequences were obtained from randomly selected clones. These analyses were repeated twice and the sum from both assays is shown in Table 1.

Table S3. CHAC1 expression induced by *H. pylori i*nfection leading to *TP53* mutation in AGS cells (Results from each of the two experiments)

	Results from each of the two experiments			
	1st experiment		2nd experiment	
	Total Mutated clones	Mutations causing amino acid substitutions	Total Mutated clones	Mutations causing amino acid substitutions
Uninfected	0 / 30	0 / 35370 (0.00 / 10^4)	0 / 30	0 / 35370 (0.00 / 10^4)
cagA (-) *H. pylori* *	0 / 29	0 / 34191 (0.00 / 10^4)	0 / 30	0 / 35370 (0.00 / 10^4)
cagA (+) *H. pylori* *	5 / 26	4 / 30654 (1.30 / 10^4)	9 / 33	4 / 38907 (1.03 / 10^4)
cagA (+) *H. pylori* + with scrambled siRNA†	5 / 29	4 / 34191 (1.17 / 10^4)	5 / 24	2 / 28296 (0.71 / 10^4)
cagA (+) *H. pylori* + with CHAC1 siRNA†	0 / 30	0 / 35370 (0.00 / 10^4)	0 / 28	0 / 33012 (0.00 / 10^4)

*Exome sequence between exon 2 and 11 of *TP53* was amplified and cloned from untreated AGS cells 16 days after *H. pylori* infection or uninfected cells as control, followed by analysis of nucleotide sequences in randomly selected clones. †Mutation frequencies for *TP53* amplified from AGS cells treated with scrambled siRNA or CHAC1 siRNA at 16 days after *H. pylori* infection. These analyses were repeated twice and the sum from both assays is shown in Table 1.

Table S4. Distribution of nucleotide alterations in the *TP53* sequence and resultant amino acid substitutions

Codon position	Base change	Amino acid change
Day4 CHAC1-WT		
78	G>A	Ala>Thr
388	A>G	Glu>Gly
Day8 CHAC1-WT		
29	A>G	Asn>Ser
33	T>C	Ser>Pro
94	T>C	Ser>Pro
245	G>A	Gly>Asp
248	G>A	Arg>Gln
351	A>G	Lys>Glu
364	C>T	Ala>Val
Day16 CHAC1-WT		
44	G>A	Met>Ile
54	T>C	Phe>Leu
75	C>A	Pro>His
122	G>A	Val>Met
168	A>G	His>Arg
176	G>A	Cys>Tyr
177	C>A	Pro>Thr
177	C>A	Pro>Thr
197	T>C	Val>Ala
244	G>A	Gly>Asp
245	G>A	Gly>Asp
253	C>T	Thr>Iso
345	A>G	Asn>Ser
cagA (+) *H. pylori*		
56	G>A	Glu>Lys
84	G>C	Ala>Pro
110	C>T	Arg>Cys
137	T>C	Leu>Pro
176	G>C	Cys>Ser
231	A>G	Thr>Ala
313	A>G	Ser>Gly
318	C>A	Pro>Thr
cagA (+) *H. pylori* + scrambled siRNA		
1	G>T	Met>Ile
66	G>A	Met>Ile
170	C>T	Thr>Met
176	G>C	Cys>Ser
251	A>G	Ile>Val
270	T>C	Phe>Leu
